# Application of Olefin Metathesis in the Synthesis of Carbo- and Heteroaromatic Compounds—Recent Advances

**DOI:** 10.3390/molecules28041680

**Published:** 2023-02-09

**Authors:** Szymon Rogalski, Cezary Pietraszuk

**Affiliations:** Faculty of Chemistry, Adam Mickiewicz University, Poznań, Uniwersytetu Poznańskiego 8, 61-614 Poznań, Poland

**Keywords:** metathesis, heteroaromatics, carboaromatics

## Abstract

The olefin metathesis reaction has found numerous applications in organic synthesis. This is due to a number of advantages, such as the tolerance of most functional groups and sterically demanding olefins. This article reviews recent advances in the application of the metathesis reaction, particularly the metathetic cyclization of dienes and enynes, in synthesis protocols leading to (hetero)aromatic compounds.

## 1. Introduction

Olefin metathesis is currently an invaluable tool for advanced organic and polymer synthesis [1,2,3]. The reaction is frequently used as a step in a sequence of processes leading to complex multifunctional molecules, such as natural products, and biologically active compounds [4]. The great importance of (hetero)arenes in nature, scientific research and the chemical industry does not require justification. Aromatic compounds are essential for the industry and vital to biochemistry [5,6]. More recent applications of aromatics include the synthesis of functional materials. The greatest interest among research groups in using various types of metathetic transformations of olefins, dienes, and enynes in organic synthesis, including the synthesis of aromatic compounds, followed by an explosion of development in the design and synthesis of well-defined alkylidene complexes of molybdenum and ruthenium catalysts of olefin metathesis. The application of olefin metathesis in the synthesis of carbo- and heteroaromatic compounds has been described in numerous reviews. The most comprehensive overview of the literature up to 2009 was provided by Otterlo and Koning [7]. The state-of-the-art of synthetic routes to carbocyclic aromatic compounds via ring-closing alkene and ene metathesis up to 2014 was reviewed by de Koning and van Otterlo [8]. In 2013, a monograph *Transition-Metal-Mediated Aromatic Ring Construction* was published with a chapter by Yoshida on olefin metathesis [9]. The work of Donohoe et al. [10] is an earlier review in this area. Race and Bower [11] described the state-of-the-art in the synthesis of heteroaromatic compounds by alkene and enyne metathesis up to and including 2014. An earlier review describing the possibilities offered by RCM in the synthesis of aromatic heterocycles was written by Donohoe, Fishlock, and Procopiou [12]. Moreover, in 2016, Potukuchi, Colomer, and Donohoe reviewed the synthesis of heteroaromatic compounds, with a focus on the achievements of their own group [13]. Reviews describing the synthesis of specific groups of compounds using metathetic transformations are indicated in the relevant sections of this review. The aim of this review is to highlight new developments in the use of olefin metathesis in the synthesis of aromatic compounds that were reported between the years 2015 and 2022. Procedures involving the de novo formation of aromatic rings, rather than the modification of aromatic starting compounds, are described.

## 2. Olefin Metathesis

**Scheme 1:**
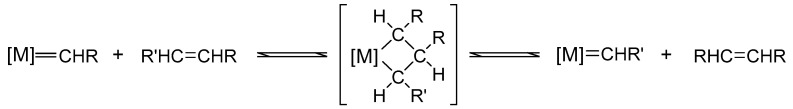
Metallacarbene mechanism of olefin metathesis.

Olefin metathesis is a catalytic transformation of olefins which involves the exchange of double bonds between carbon atoms. The reaction is catalyzed by alkylidene complexes, and its mechanism consists of a sequence of cycloaddition and productive cycloreversion steps proceeding via metallacyclobutane intermediates (Scheme 1) [14].

**Scheme 2:**
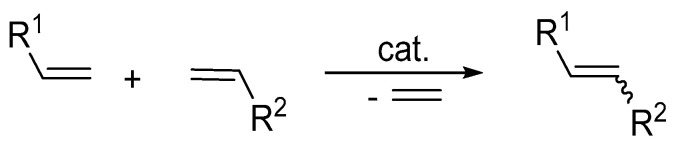
Olefin cross-metathesis (CM).

**Scheme 3:**
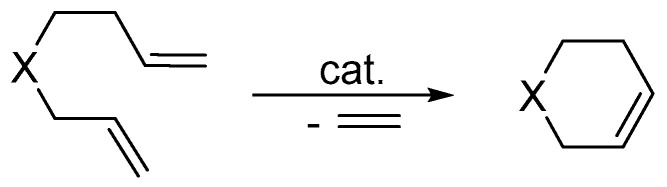
Ring-closing olefin metathesis (RCM).

Of the many types of olefin metathesis, the most useful in organic synthesis are the cross-metathesis of olefins (CM) and the metathetic cyclization of dienes (RCM) (Scheme 2 and Scheme 3).

**Scheme 4:**
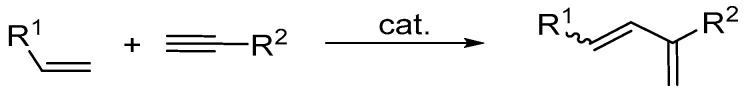
Ene-yne cross-metathesis (EYCM).

**Scheme 5:**
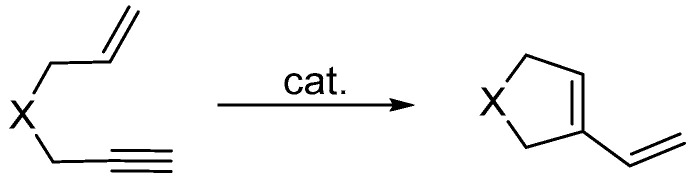
Ring-closing enyne metathesis (RCEM).

The mechanism of olefin metathesis also allows for transformations involving triple bonds, i.e., ene-yne cross-metathesis (EYCM) and enyne ring-closing metathesis (RCEM) (Scheme 4 and Scheme 5, respectively).

Metathetic transformations of the triple bond can lead to a mixture of regio- and stereoisomers [15]. Fortunately, in the case of RCEM, the synthesis of small and medium rings in the presence of ruthenium catalysts selectively leads to the formation of an exocyclic product, as shown in Scheme 5 [16]. RCEM should not be confused with skeletal reorganization, which is catalyzed by late-transition metals, such as Pd, Pt, Ru, Au, Ir, and Rh, and can lead to similar products but does not follow the metallacarbene mechanism [17,18]. EYCM may also be used for the synthesis of 1,3-dienes. Although the reaction can lead to the formation of stereo- and regioisomers, a number of examples of the selective course of the reaction have been described [19,20,21]. Cross-metathesis between a 1-alkene and a 1-alkyne leads to 1,3-substituted 1,3-dienes, as shown in Scheme 4. Moreover, the CM of internal alkynes with ethene selectively leads to the formation of 2,3-substituted 1,3-butadienes. Metathetic transformations involving triple bonds are characterized by total atom efficiency.

**Figure 1:**
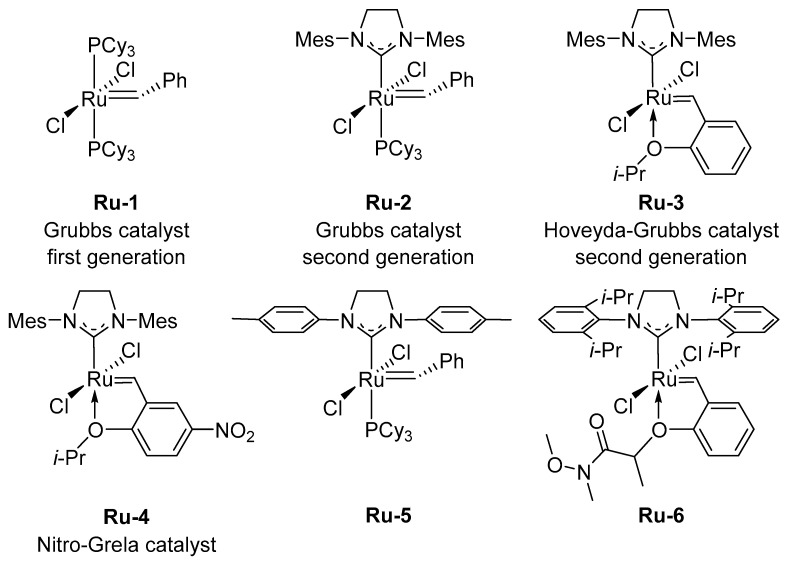
Ruthenium-based olefin metathesis catalysts referred to in this review.

The olefin metathesis has become an important tool in the synthesis of organic compounds. This is a consequence of the dynamic progress in the synthesis and design of well-defined catalysts such as the alkylidene complexes of tungsten, molybdenum and ruthenium. Especially the family of ruthenium-based catalysts tolerant of normal organic and polymer processing conditions and preserving their catalytic properties in water and in the presence of the majority of functional groups has enabled a great number of applications in organic (and polymer) synthesis (see [22], for example). Tungsten and molybdenum complexes, including the most commonly used, commercially available, highly active molybdenum complex, [(NAr){OC(CH_3_)(CF_3_)_2_}_2_M=CH(2,6-*i*-Pr_2_C_6_H_3_)] (Mo-1) [23], suffer from the high oxophilicity of the metal centers, making them extremely sensitive to oxygen, moisture, and numerous functional groups [22]. Although many well-defined ruthenium olefin metathesis catalysts have been described, most of the literature procedures use only a few commercially available complexes (Ru-1–Ru-3, Figure 1).

Olefin metathesis has gained a number of applications in advanced organic synthesis, chemical biology, medicinal chemistry, the synthesis of simple molecules, and the total synthesis of natural products [1,2,3,4].

## 3. Synthesis of Aromatic Heterocycles

Olefin metathesis, especially ring-closing metathesis (RCM), has been extensively used in the synthesis of heteroaromatic compounds of divergent types. It has been exploited efficiently as a key step for the preparation of the pyrroles, furans, pyridines, imidazoles, and related systems. All of these compounds are largely important as structural units in natural products and are used as synthons in further transformations. In this section, different approaches to the synthesis of heteroaromatic compounds using olefin metathesis as a key step are reviewed. The syntheses of heteroaromatics involving olefin metathesis as one of the key steps has been summarized in several reviews [24,25,26,27].

This section presents various strategies for the synthesis of heteroaromatic compounds by using olefin metathesis as a key step. Knowledge on the synthesis of heteroaromatic compounds involving olefin metathesis as one of the key steps has been summarized in several review papers [24,25,26,27]. In the synthesis of (hetero)aromatic compounds, the metathesis step is used in a sequence of reactions involving the formation of a heterocyclic ring by RCM of a diene or enyne and its subsequent aromatization.

### 3.1. Synthesis of Five-Membered Rings

**Scheme 6:**
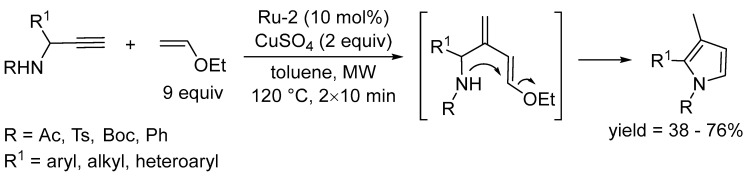
Synthesis of 1,2,3-substituted pyrroles.

Since pyrrole and furan synthons are commonly present in many pharmaceuticals and biologically active molecules, the efficient synthesis of these compounds continues to be of interest to organic chemists. In 2015, Castagnolo and co-workers described an elegant methodology that represents the first example of one-pot synthesis of substituted pyrroles via enyne cross-metathesis (CM) cyclization reaction, using propargylamines and ethyl vinyl ether as reagents. The described CM/cyclization protocol is performed in the presence of Ru-2, is microwave-assisted, and offers a convenient approach to the synthetically challenging 1,2,3-substituted pyrroles (Scheme 6) [28].

**Scheme 7:**
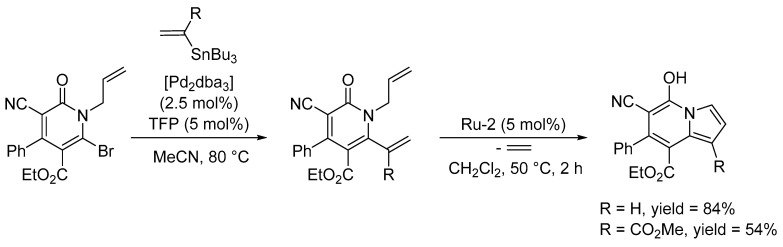
Synthesis of indolizin-5-ol derivatives.

In 2015, Spring reported the development of a new strategy for the synthesis of biologically interesting indolizin-5(3*H*)-ones [29]. Performed experiments showed that indolizin-5(3*H*)-ones are a relatively unstable class of compounds and have a tendency to tautomerize to indolizin-5-ols. This observation was further exploited to prepare other useful compounds based on 6,5-azabicyclic scaffolds, which are difficult to obtain with the use of typical methods. The authors developed a procedure based on the ring-closing metathesis reaction effectively applied to construct the azabicyclic heteroaromatic ring system present in indolizin-5-ols (Scheme 7).

The proposed strategy represents an unprecedented approach toward this type of scaffold. Several heteroaromatic derivatives were obtained with good-to-high yields (50–84%). The procedure is step-efficient, and a number of novel analogues could be readily accessed through the adaptation of the substituents around the heterocyclic core in the starting pyridine molecule.

Obtained indolizin-5(3*H*)-ones and indolizin-5-ols are relatively rare; therefore, they can potentially be of interest as new synthons in drug and agrochemical synthesis.

**Scheme 8:**
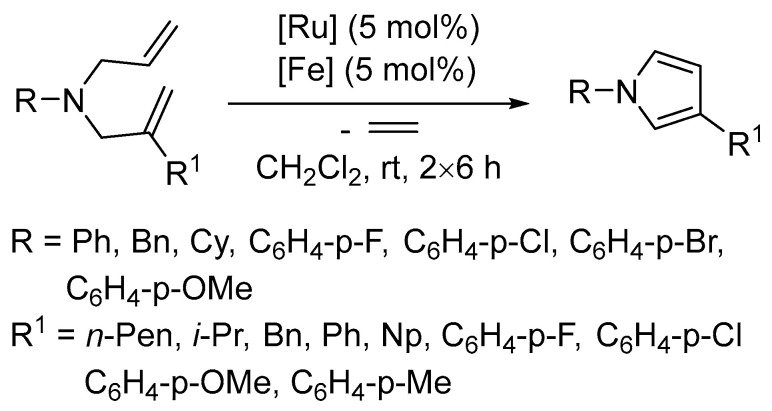
Synthesis of substituted pyrroles via Ru-catalyzed RCM and Fe-catalyzed aromatization.

Another route to pyrroles substituted in the *β*-position was developed by Samec and co-workers, using four high-yielding catalytic steps in the presence of Pd, Ru, and Fe catalysts [30]. The authors described a Pd-catalyzed effective method for the synthesis of unsymmetrical diallylated aromatic amines. In the final key step of the procedure, Ru-Catalyzed RCM, followed by aromatization in the presence of Fe complexes, was employed (Scheme 8).

The method is atom-efficient, with the formation of water and ethene as side-products. The procedure is general and gives pyrroles substituted in the *β*-position with alkyl, benzyl, or aryl groups with good overall yields. 

**Scheme 9:**
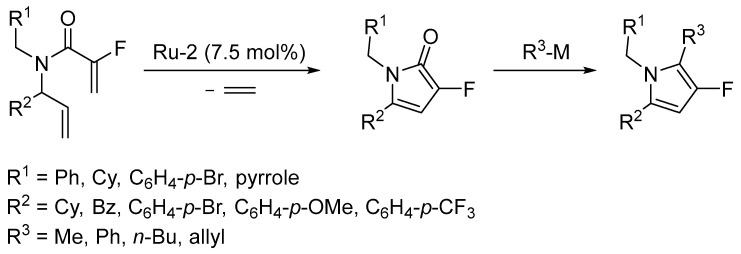
Synthesis of fluorinated polysubstituted pyrroles.

An efficient and selective approach for the synthesis of polyfunctionalized 3-fluoropyrroles starting from commercial aldehydes was described by Marquez and co-workers [31]. The key step of the described methodology is RCM of diene in the presence of Ru-2, followed by an aromatization process based on the alkylation of the obtained fluorolactams with methyllithium. The procedure proceeded smoothly to generate the desired pyrrole units in excellent yield (73–92%). The authors proposed that the aromatization process in the presence of MeLi proceeded through the formation of a hemiaminal, followed by the elimination of water and the isomerization of double bonds (Scheme 9).

**Scheme 10:**
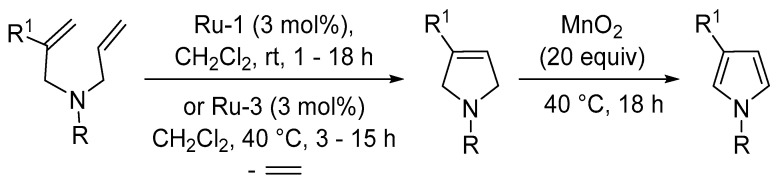
One-pot RCM-oxidation for the synthesis of substituted pyrroles.

*N*-sulfonyl pyrroles were synthesized via the combination of ring-closing, or enyne metathesis, with oxidation [32]. A range of different *N*-substituted diallyl amines were subjected to RCM with Grubbs catalyst. After the first metathetical step, heterogeneous MnO_2_ was used as an effective oxidant (Scheme 10). Reasonable-to-good yields were obtained for a variety of substituted amines even when the process was performed in one-pot procedure.

**Scheme 11:**
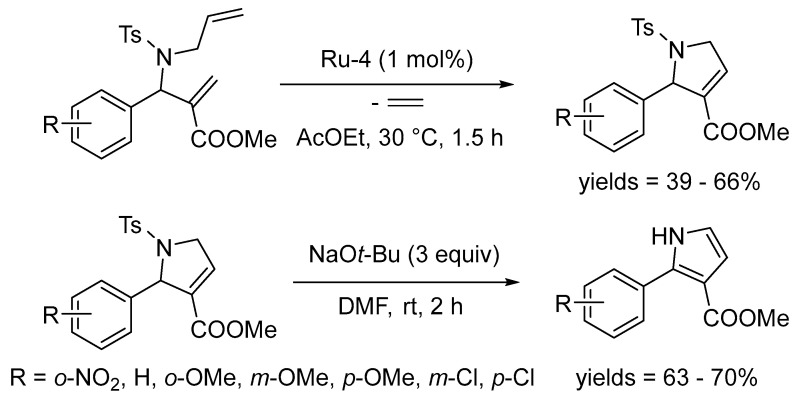
RCM/deprotection–aromatization sequence for the preparation of pyrroles.

Lamaty and co-workers described an easily scalable pyrrole synthesis strategy involving RCM in the first step and subsequent aromatization by base-induced nitrogen deprotection (Scheme 11) [33].

The authors developed the procedure of synthesis of 2-aryl-1*H*-pyrrole-3-carboxylates, using ring-closing metathesis of the corresponding *β*-amino esters as a key step. In a two-step procedure, the described methodology allowed for an efficient formation of 2-aryl substituted pyrroles, especially unprecedented 2-aryl-1*H*-pyrrole-3-carboxylates. The products were obtained in good yields, ranging from 63 to 70%, in the presence of the relatively low loading (1 mol%) of Ru-4 (Figure 1) in green solvent, such as ethyl acetate, except for DCM.

**Scheme 12:**
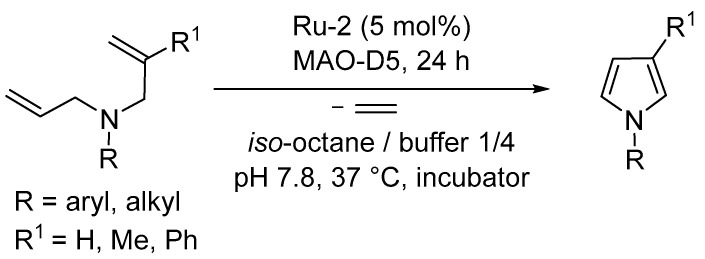
Chemoenzymatic synthesis of pyrroles.

In 2017, the Castagnolo group reported the first chemoenzymatic cascade process for the sustainable preparation of substituted pyrroles in which the ring-closing metathesis reaction was applied for the preparation of 3-pyrrolines [34]. The protocol used, for the first time, the unexplored aromatizing activity of monoamine oxidase enzymes (MAO-N and 6-HDNO), which are able to convert a wide range of *N*-aryl- and *N*-alkyl-3-pyrrolines into pyrroles under mild conditions and in high yields (Scheme 12). The authors showed that MAO-N can work in combination with Ru-2, leading to the formation of a series of substituted pyrroles derived from diallylamines, as well as anilines, in a one-pot metathesis−aromatization sequence.

**Scheme 13:**
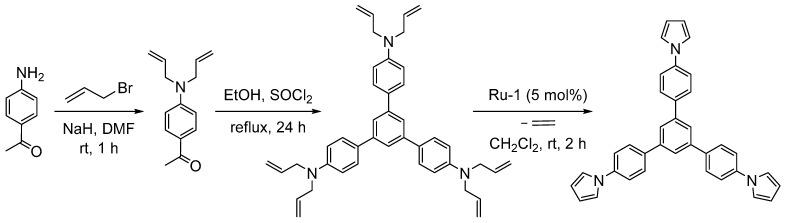
Synthesis of star-shaped pyrrole derivative via RCM key step.

Kotha and co-workers demonstrated three divergent synthetic strategies to construct pyrrole-based *C_3_*-symmetric molecule [35]. One of the synthetic protocols involved ring-closing metathesis as a key step in the presence of Ru-1. The star-shaped pyrrole derivative with *C_3_*-symmetry was prepared in good yield (84%), without the involvement of additional reagents, in one-pot RCM and a subsequent aromatization protocol, starting from hexa-allyl derivative. The hexa-allyl substituted compound could be synthesized from the corresponding di-allyl ketone by trimerization, starting from the readily available 4-aminoacetophenone (Scheme 13).

The described facile RCM/dehydrogenation (aromatization) sequence was preceded by the non-metathetic behavior of the Grubbs catalyst previously reported by the authors. An earlier approach to pyrroles involving RCM required a separate step for aromatization. Thus, the approach reported by Kotha, which does not require additional aromatization step, is more sustainable.

**Scheme 14:**
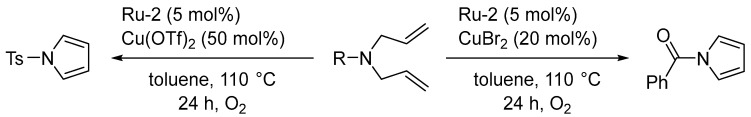
RCM/dehydrogenation sequence of sulfonyl- and acyldiallylamines.

Wu and Li proposed another convenient method for preparation of *N*-sulfonyl- and *N*-acylpyrroles via the olefin ring-closing metathesis of diallylamines in the presence of Ru-2 combined with in situ oxidative aromatization with atmospheric O_2_ and suitable copper catalyst such as Cu(OTf)_2_ or CuBr_2_ (Scheme 14) [36]. The reaction was performed via a one-pot tandem RCM/dehydrogenation procedure and afforded *N*-sulfonyl- and *N*-acylpyrroles with moderate-to-good yields (45–93%).

**Scheme 15:**
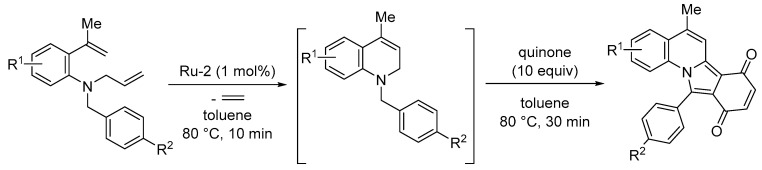
One-pot RCM/oxidation/1,3-dipolar cycloaddition protocol.

**Figure 2:**
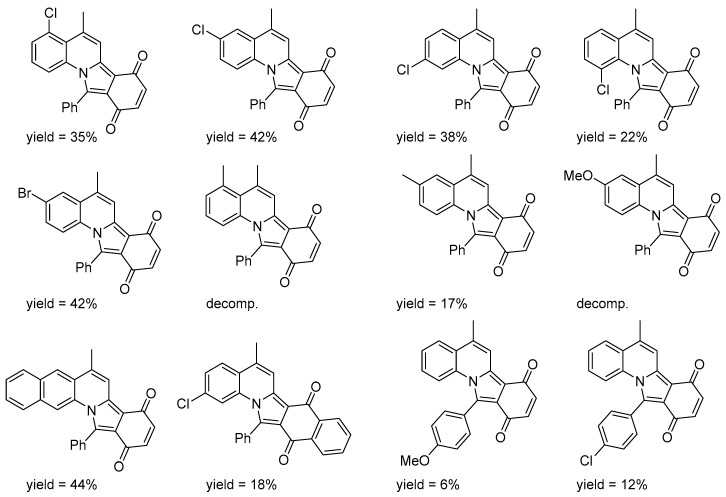
Scope of products obtained by one-pot RCM/oxidation/1,3-dipolar cycloaddition protocol.

Arisawa and co-workers synthesized 5-methylisoindolo[2,1-*a*]quinoline derivatives as novel near-infrared absorption dyes [37]. The authors applied a previously developed one-pot ring-closing metathesis (RCM)/oxidation/1,3-dipolar cycloaddition cascade protocol to prepare various isoindolo[2,1-*a*]quinoline derivatives from substituted *N*-allyl-*N*-benzylanilines (Scheme 15 and Figure 2). They proposed that the key intermediate in this reaction is the azomethine ylide derived from 1,2-dihydroquinoline.

The first RCM step was performed effectively in the presence of Ru-2 during 10 min, with relatively low catalyst loading (1 mol%) giving the intermediates nearly quantitative yields. Unfortunately, the electronic effects of substituents on the aromatic ring were responsible for the low substrate reactivity during the 1,3-dipolar cycloaddition step. It was observed that substrates bearing an electron-withdrawing group in the aromatic ring afforded higher yields in the 1,3-dipolar cycloaddition, thus giving the target molecules overall yields ranging from 22 to 55%.

In order to understand a structure and absorption−wavelength relationships, the authors performed calculations by using a time-dependent density functional theory (TD-DFT).

**Scheme 16:**
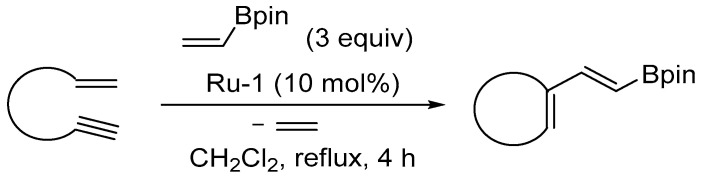
Synthesis of cyclic dienyl boronic esters via an RCEM/CM sequence.

Whiting and Carboni showed a different approach to the synthesis of pyrroles [38]. In the first step, they performed ring-closing enyne metathesis (RCEM), followed by cross-metathesis (CM) with vinylboronic esters to form a large spectrum of cyclic dienyl boronic esters (Scheme 16).

**Scheme 17:**
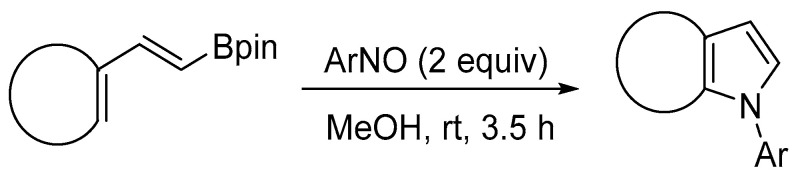
Synthesis of fused pyrroles from cyclic dienyl boronic esters.

Second step involved a nitroso-Diels–Alder reaction between a series of previously obtained dienyl boronic esters and nitrosoarenes with the formation of a heteroaromatic ring (Scheme 17).

The authors developed a new and efficient route to a series of cyclic 1,3-dienyl boronic esters via diene or enyne metathesis that were further transformed with good yields to fused pyrroles, using a one-pot nitroso-Diels−Alder/ring contraction sequence.

**Scheme 18:**
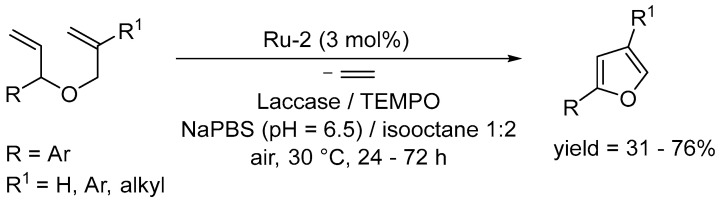
One-pot chemoenzymatic cascade for the synthesis of furans from diallyl ethers.

The unprecedented cascade procedure based on the Grubbs-catalyzed ring-closing metathesis of diallyl ethers, followed by laccase/TEMPO-catalyzed aromatization, was successfully developed by Castagnolo (Scheme 18) [39]. The catalytic activity of the Trametes versicolor laccase/TEMPO system was demonstrated for the first time in the aromatization of 2,5-dihydrofurans to the corresponding furans. The synthesis of furans from diallylethers was performed in moderate-to-high yields (31–76%), using a two-step RCM process in the presence of Ru-2 and subsequent laccase/TEMPO-catalyzed chemo-enzymatic aromatization.

**Scheme 19:**
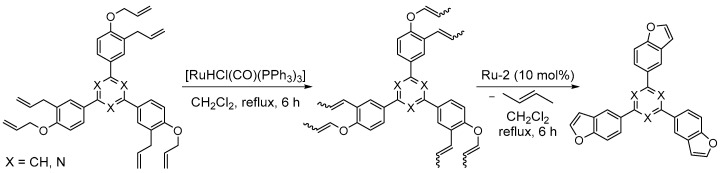
Synthesis of star-shaped phenyl or triazine central core benzofuran derivatives via isomerization and RCM sequence.

Kotha reported a new synthetic strategy leading to *C_3_*-symmetric star-shaped phenyl and triazine central cores bearing oxepine and benzofuran ring systems [40]. The authors exploited a three-step procedure to construct *C_3_*-symmetric molecules, starting with the commercially available *p*-hydroxyacetophenone and *p*-hydroxybenzonitrile, through cyclotrimerization, double-bond isomerization, and ring-closing metathesis sequence as key steps. A series of star-shaped allyl-ether derivatives were obtained through the sequence: cyclotrimerization, Claisen rearrangement and allylation. Then allyl substituted ethers were subjected to Ru-hydride-catalyzed double-bond isomerization performed in toluene at 80 °C. The reaction resulted in the formation of a double-bond isomerized product with 91% yield as a mixture of diastereomers. The obtained compounds were subjected to metathesis with a second-generation Grubbs catalyst in methylene chloride at 40 °C to give *C_3_*-symmetric star-shaped phenyl and triazine core derivatives bearing benzofuran moieties with yields of the metathesis step reaching 80% (Scheme 19).

All the compounds obtained therein showed moderate-to-strong fluorescence, with a strong emission band around 325–350 nm. The described methodology is the first successful attempt to introduce oxepine ring systems into *C_3_*-symmetric star-shaped derivatives of a phenyl or triazine core by means of an olefin metathesis reaction.

**Scheme 20:**
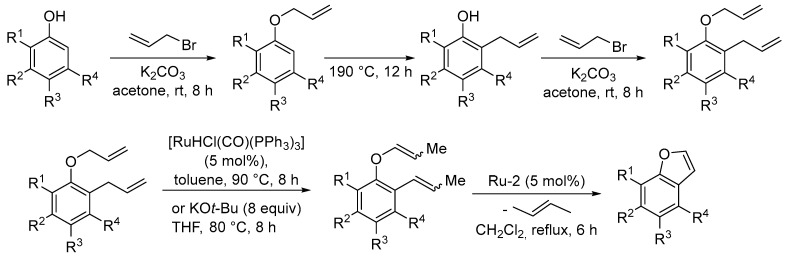
Synthesis of benzofurans from variously substituted phenols.

A multistep strategy involving the synthesis of benzofurans, 2*H*-chromenes, and benzoxepines from various phenols was recently proposed by Kotha (Scheme 20) [41]. The described procedure employed Claisen rearrangement and followed by RCM as key steps. At first, a number of substituted allyl aryl ethers were synthesized by alkylation of corresponding phenols with allyl bromide in the presence of potassium carbonate. The resulting allyl aryl ethers were then subjected to a Claisen rearrangement in the absence of a solvent to produce allylphenols, which were then converted to diallyl compounds by *O*-allylation in 97–98% yield.

**Scheme 21:**
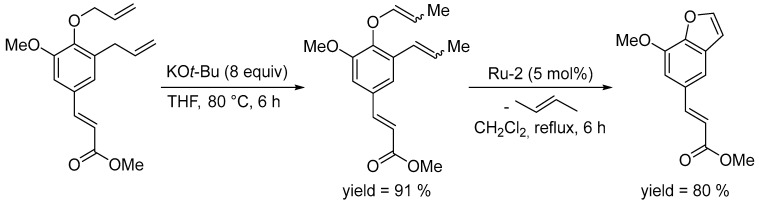
Synthesis of 7-methoxywutaifuranate.

The authors then carried out a standard isomerization reaction catalyzed by [RuHCl(CO)(PPh_3_)_3_] and investigated an alternative method of double-bond migration, using potassium tert-butoxide (KO*t*-Bu) in THF. Isomerization products were obtained as a mixture of isomers in 89–94% yields. The final step in the construction of the heteroaromatic ring involved an RCM step, providing the corresponding benzofurans, with a yield of 84–85%. Using the proposed procedure, a wide range of products differing in the substituents on the 6-membered ring was obtained. In addition, naturally occurring benzofurans, such as 7-methoxywutaifuranate, were also synthesized with good yields (80%), using the described protocol (Scheme 21).

**Scheme 22:**
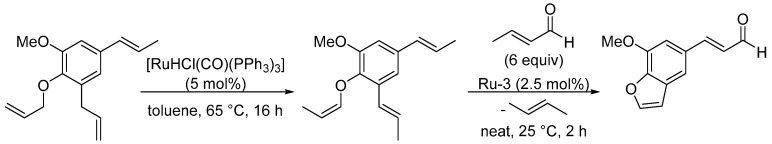
Key isomerization and metathesis steps in the synthesis of 7-methoxywutaifuranal.

Another interesting example of a multistep synthesis of 7-methoxywutaifural using olefin metathesis was recently reported by Schmidt and co-workers [42]. The authors showed that isoeugenol can undergo efficient cross-metathesis with acrolein and crotonaldehyde in the presence of Hoveyda–Grubbs catalyst (Ru-3). This observation was used in the multistep synthesis of the natural product-7-methoxywutaifuranal (Scheme 22).

### 3.2. Synthesis of Six-Membered Rings

Six-membered ring heteroaromatics, such as pyridines and pyridazines, are useful scaffolds that are present in various drugs and natural products. This section reviews strategies for the synthesis of six-membered heteroaromatics that use RCM as a key step. Progress in the synthesis of azonic aromatic heterocycles was summarized by Vaquero [43].

**Scheme 23:**
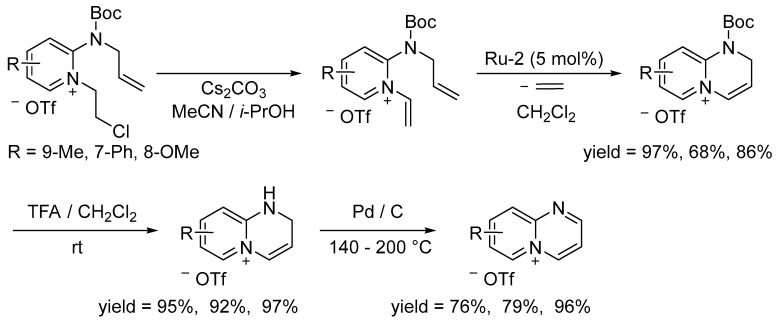
Application of RCM for the construction of the pyrimidinium ring.

**Scheme 24:**
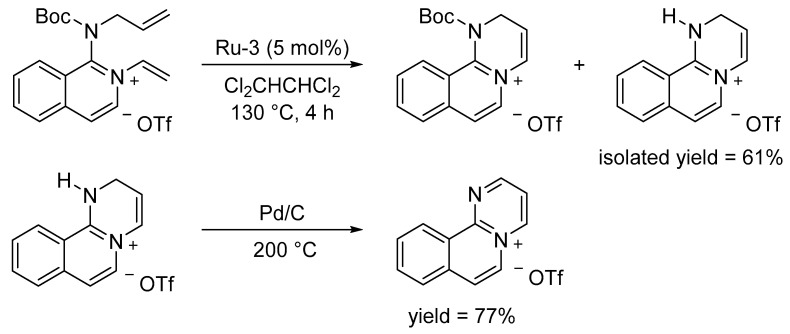
Synthesis of pyrimido[2,1-*a*]isoquinolinium, using RCM/aromatization sequence.

In 2015, Cuadro and Vaquero reported new approach to synthesize the 1-azaquinolizinium (pyrido[1,2-*a*]pyrimidin-5-ium) and its derivatives based on a ring-closing metathesis reaction of pyridinium azadienes in the presence of the second-generation Grubbs catalyst (Ru-2) or Hoveyda–Grubbs catalyst (Ru-3) (Scheme 23) [44]. The described strategy was also successfully applied to the unprecedented synthesis of benzo-1-azaquinolizinium (pyrimido[2,1-*a*]isoquinolinium) cation by RCM (Scheme 24). The proposed synthetic route involved the formation of the new C3–C4 bond to give the pyrimidinium ring. The authors prepared protected pyridinium azadienes derivative with the use of protection/allylation/dehydrohalogenation cascade, starting from commercially available 2-aminopyridine. Then obtained diene was subjected to RCM, affording the expected product. The subsequent deprotection of the RCM product gave the corresponding 1,2-dihydro-1-azaquinolizinium triflate, which was further dehydrogenated by heating at 200 °C in the presence of Pd/C (10%) without solvent to give the 1-azaquinolizinium triflate.

**Scheme 25:**
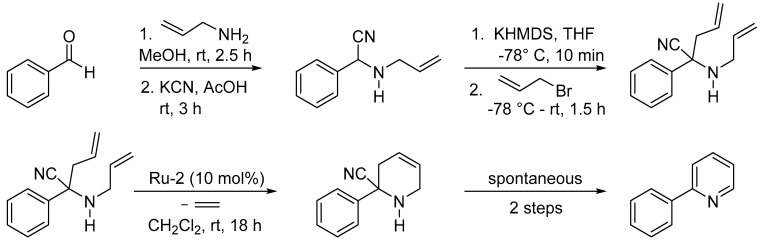
Synthesis of phenylpyridines, using RCM as a key step.

A simple three-step synthesis of substituted pyridines based on an alkylation/ring-closing metathesis/aromatization sequence was described by Opatz and colleagues [45]. The synthetic strategy involved the preparation of diallylic *α*-aminonitrile in a two-step approach. At first Strecker reaction starting from benzaldehyde, allylamine, and potassium cyanide was performed to afford 2-(allylamino)-2-phenylacetonitrile. Then deprotonation with the use of KHMDS, followed by alkylation with allyl bromide, was applied to give corresponding diallyl-substituted α-aminonitrile. Finally, the RCM of unprotected allylic amine and subsequent spontaneous dehydrocyanation and oxidation were employed as the key steps of the developed sequence (Scheme 25). The desired substituted pyridines were obtained with moderate yields ranging from 24 to 48%.

Suresh studied [2+2+2] cyclotrimerization of diynes and nitriles leading to efficient synthesis of pyridine and its derivatives by DFT calculations. It was shown that two mechanisms of the reaction are plausible—metallacarbene mechanism of metathesis and non-metallacarbene route. The strategy can be potentially used in the total synthesis of natural products [46].

**Scheme 26:**
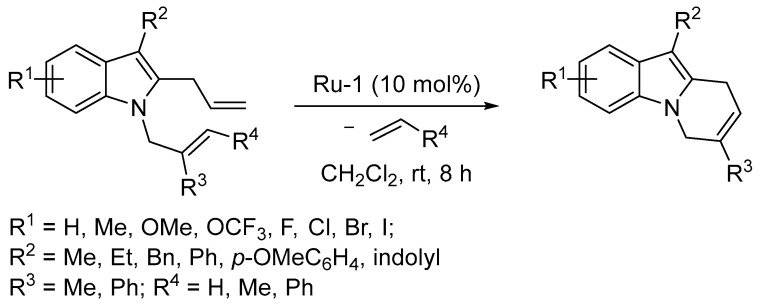
RCM of 1,2-diallylindoles.

Dash and co-workers reported the addition of Grignard reagent to oxindoles. This previously unexplored strategy represented a regiospecific approach to 2- and 2,3-disubstituted indole derivatives obtained via a one-pot aromatization driven dehydration pathway [47]. The described method allowed for the convenient preparation of diallylindoles, which can be used as ring-closing-metathesis (RCM) precursors for the orthogonal synthesis of pyridyl[1,2-*a*]indoles (Scheme 26).

**Scheme 27:**
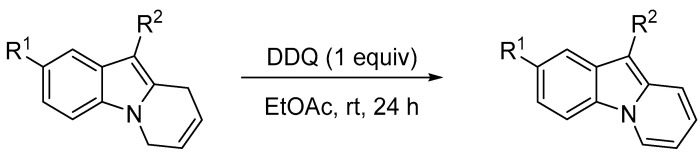
DDQ-mediated aromatization of dihydropyridoindoles.

**Figure 3:**
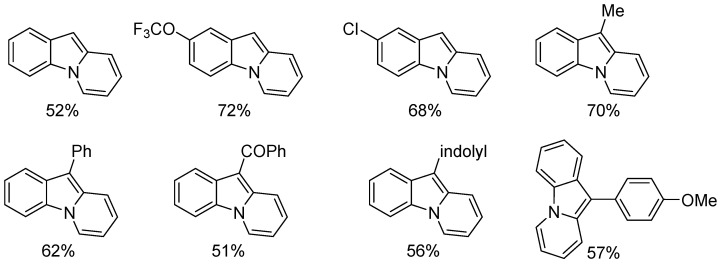
Scope of pyridoindoles synthesized via RCM/aromatization sequence.

The authors synthesized a variety of *N*-allyl 2-oxindoles and effectively transformed them to *N*-substituted 2-allyl indole derivatives by treatment with allylmagnesium bromide in yields up to 86%. Then the diallylindoles were used for the orthogonal synthesis of conjugated nitrogen-containing heterocycles, using RCM. In this approach, a series of 1,2-diallyl indolyl derivatives were subjected to metathesis cyclization and yielded the desired substituted dihydropyridylindoles in good yields (72–86%). Finally, dihydropyridoindoles were effectively transformed into aromatic derivatives via a DDQ-mediated oxidative aromatization protocol (Scheme 27 and Figure 3).

**Scheme 28:**
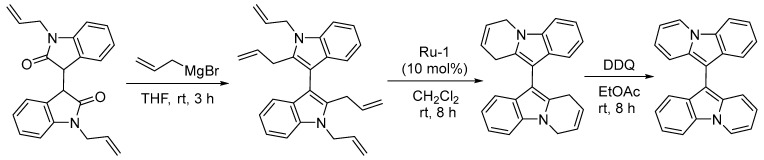
Synthesis of dimeric pyridoindoles.

The optimized reaction sequence was employed in the synthesis of dimeric pyridoindoles (Scheme 28)

## 4. Synthesis of Aromatic Carbocycles

**Scheme 29:**
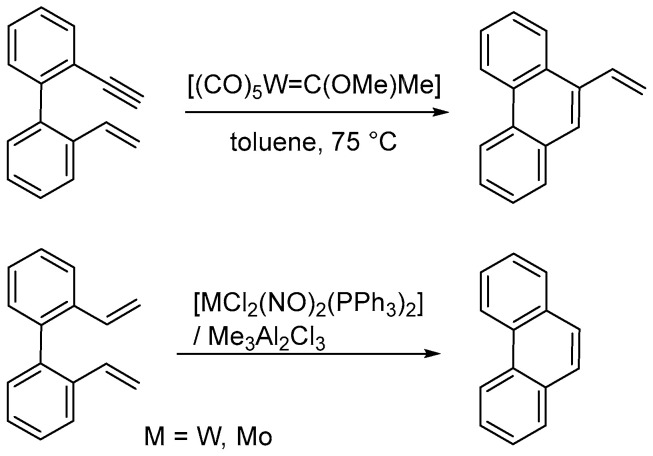
Direct formation of aromatic compounds via RCEM or RCM.

Dienes or enynes RCM can lead directly to the formation of new aromatic ring systems. Such a strategy has been used in the synthesis of phenanthrenes (Scheme 29) [48,49].

**Scheme 30:**
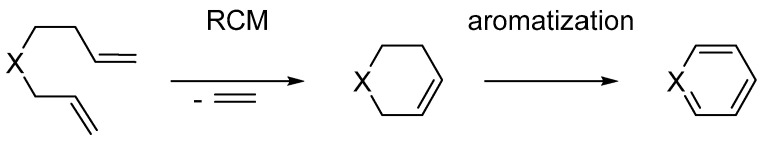
A strategy for the synthesis of aromatic rings, RCM/aromatization sequence.

Metathesis cyclization, however, does not usually lead to the formation of an aromatic ring. Thus, the synthesis of (hetero)aromatic compounds uses a metathesis step in a sequence of reactions involving the formation of a carbo- or heterocyclic ring and its subsequent aromatization. The sequence using the diene RCM step is shown in Scheme 30.

**Scheme 31:**
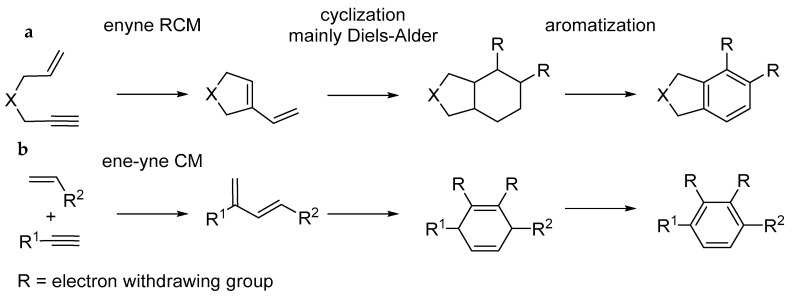
A strategy for the synthesis of aromatic compounds. Reaction sequence, RCEM (**a**), or EYCM; (**b**) Diels–Alder cycloaddition–aromatization.

RCM enynes (RCEM) and ene-yne cross-metathesis (Scheme 31) are used in a similar manner. 

### 4.1. Synthesis of Aromatic Carbocycles Fused to Heterocyclic Rings

Recently, Dash overviewed applications of RCM for the synthesis of carbazoles and indole-fused heterocycles [50].

**Scheme 32:**
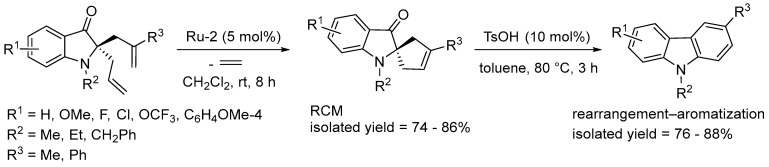
A strategy for the synthesis of carbazoles from isatins based on RCM and ring-rearrangement aromatization.

A new method for the synthesis of carbazoles via ring-closing metathesis of 2,2-diallyl-3-oxindoles, followed by ring-rearrangement aromatization of the resulting spirocyclic indoles, was proposed by Dash and co-workers (Scheme 32) [51]. The method is based on the novel ring-rearrangement aromatization that was observed when spirocyclic indoles were treated with Bronsted acids (TsOH, TfOH, and TFA) in hot toluene (80 °C).

**Scheme 33:**
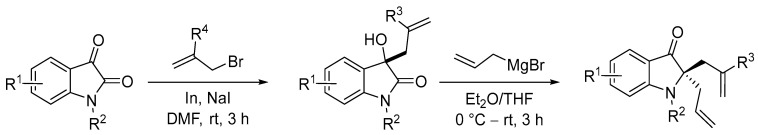
Introduction of two different allyl groups into the isatin molecule.

**Scheme 34:**
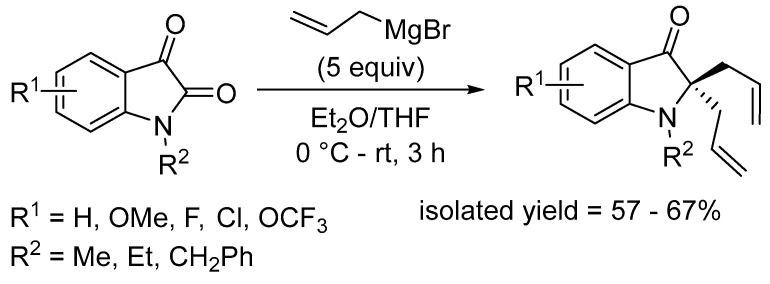
Addition of allylmagnesium bromide to *N*-substituted isatins.

Key substrates were prepared by addition of an excess of allylmagnesium bromide to *N*-substituted isatins (Scheme 33 and Scheme 34).

**Scheme 35:**
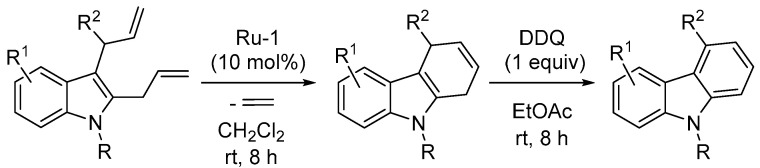
Synthesis of substituted carbazoles.

The procedure enables the convenient synthesis of carbazole from isatin derivatives and can be successfully used for the synthesis of carbazole natural products. Continued research on the addition of the allylic Grignard reagent to oxindoles resulted in the development of a procedure to obtain 2-substituted and 2,3-disubstituted indoles via a one-pot dehydrative aromatization protocol [47]. The synthesized 2,3-diallyl indoles were used to synthesize carbazole derivatives via RCM and aromatization sequence (Scheme 35).

The addition of Grignard reagents to substituted 2-oxoindoles was proven to be a tool for generating nitrogen-containing heterocycles by using simple organic transformations. This strategy was used in the synthesis of various substituted carbazoles, as well as several naturally occurring carbazole alkaloids.

**Figure 4:**
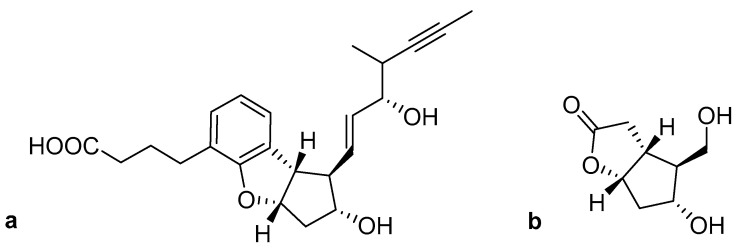
Structures of Beraprost (**a**) and (-)-Corey lactone diol (**b**).

The ring-closing metathesis reaction and aromatization were proposed as key steps in the formation of the tricyclic backbone of cyclopenta[*b*]benzofuran, a key intermediate of Beraprost (a prostacyclin analog) (Figure 4a), starting with (-)-Corey lactone diol (Figure 4b) [52].

**Scheme 36:**
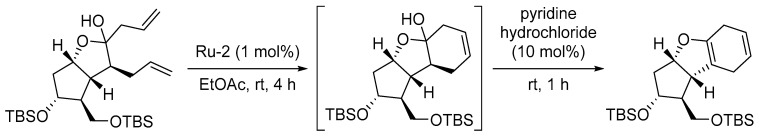
RCM of diene lactol, followed by spontaneous formation of cyclic diene.

RCM proceeded efficiently in the presence of a second-generation Grubbs catalyst under mild conditions and was followed by dehydration step performed in the presence of pyridine hydrochloride (Scheme 36).

**Scheme 37:**
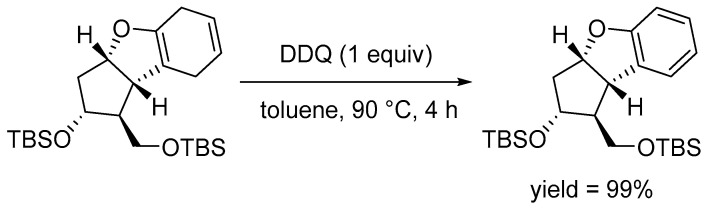
Synthesis of benzofuran skeleton via oxidative aromatization.

The benzofuran core was obtained by dehydrogenative aromatization of diene ring, using 2,3-dicyano-5,6-dichlorobenzoquinone (DDQ) (Scheme 37).

**Scheme 38:**
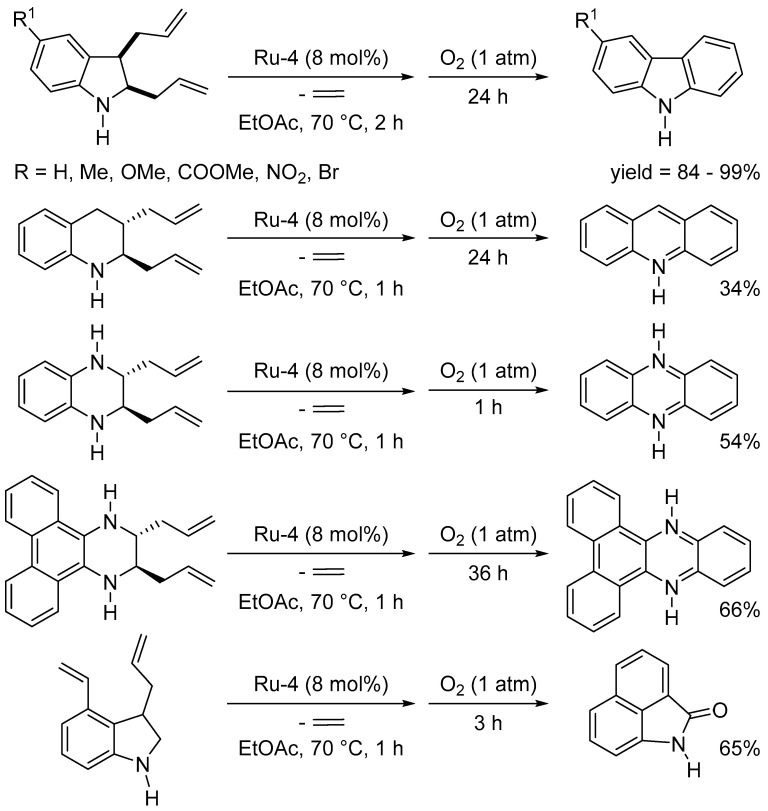
Scope of the assisted-tandem RCM/aerobic dehydrogenative aromatization. The optimal reaction conditions are indicated.

Tokuyama and co-workers reported the catalytic activity of ruthenium alkylidene complexes in the aerobic catalytic dehydrogenation of nitrogen-containing heterocycles with dioxygen as an oxidant [53]. The reaction can be used for the dehydrogenative aromatization of various nitrogen-containing heterocycles. Moreover, using the catalytic activity of alkylidene ruthenium complexes in the RCM of dienes, the authors developed the novel assisted tandem catalysis, using the ring-closing metathesis and dehydrogenative aromatization sequence (Scheme 38). Molecular oxygen was used as a trigger and an oxidation agent. Conditions to enable the highly efficient course of the process were proposed. The procedure involves the use of a ruthenium alkylidene complex Ru-5 (Figure 1), which is a modified version of the Ru-2 that bears less steric substituents at the nitrogen atoms of the imidazol-2-ylidene ligand. This catalyst was found to be optimal for the RCM/aerobic oxidation sequence.

The usefulness of the Tokuyama procedure was demonstrated by its application to the synthesis of the natural antibiotic pyocyanin.

### 4.2. Synthesis of Benzene Derivatives

Most of synthetic effort in the synthesis of simple hetero- and carboarenes via metathesis was performed during the huge development of olefin metathesis applications in organic synthesis and is described in previous reviews [7,8,10]. Currently, the research intensity, as measured by the number of publications, is much lower, and research groups are focusing on using metathesis to obtain more complex systems.

**Scheme 39:**
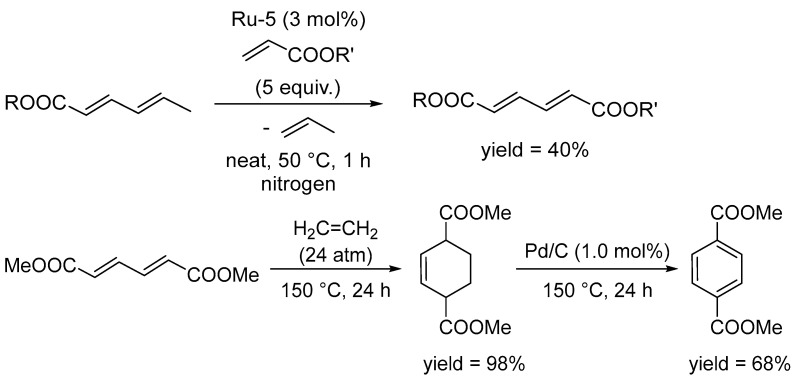
A one-pot, two-step process for the synthesis of dimethyl terephthalate based on the Diels–Alder reaction of dimethyl muconate with ethylene and subsequent aromatization in the presence of a Pd/C catalyst.

Lobo and co-workers reported a procedure for obtaining terephthalates, which includes the use of the CM, Diels–Alder cycloaddition and dehydrogenative aromatization sequence (Scheme 39) [54]. The proposed procedure is eco-friendly in nature due to the use of fully biodegradable reagents (sorbate and acrylic esters).

The highest activity of the cross-metathesis step was achieved in the presence of modified Hoveyda–Grubbs catalyst Ru-6 (Figure 1).

**Scheme 40:**
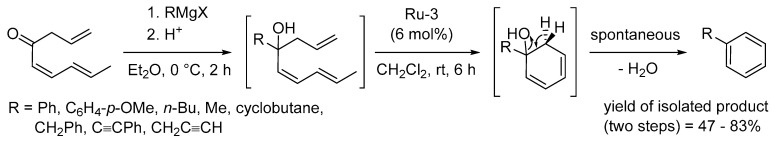
Synthesis of monosubstituted aromatic ring via ring-closing metathesis, followed by spontaneous 1,2-elimination.

Ward described a procedure of the generation of monosubstituted aromatic rings that uses a ring-closing metathesis and spontaneous 1,2-elimination sequence (Scheme 40) [55]. The method enables the synthesis of benzene derivatives with moderate-to-high yields. The advantage of the procedure is that there is no need to use strong oxidants in the aromatization step.

**Scheme 41:**
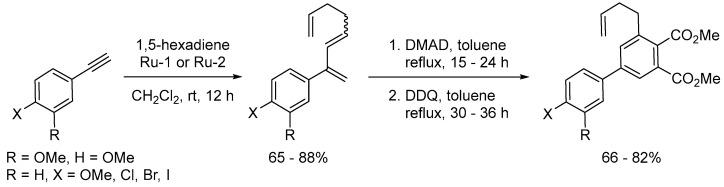
Strategy for the synthesis of biphenyls from arylalkynes.

**Scheme 42:**
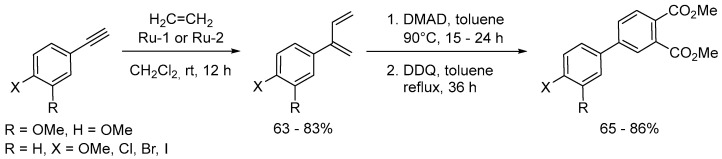
Strategy for the synthesis of biphenyls from arylalkynes. Ethene was used as the olefinic reaction partner in ene-yne CM.

The sequence ene-yne cross-metathesis, Diels−Alder cycloaddition, and aromatization were applied to synthesize a series of biaryls, starting from alkynes [56]. In the ene-yne cross-metathesis step, monosubstituted phenylacetylenes were treated with 1,5-hexadiene (Scheme 41) or ethylene (Scheme 42) in the presence of a Grubbs first- or a second-generation catalyst. Cycloaddition and subsequent aromatization afforded the desired biphenyls in good yields. Two exemplary ethynylated amino acid derivatives were also shown to undergo the proposed sequence.

**Scheme 43:**
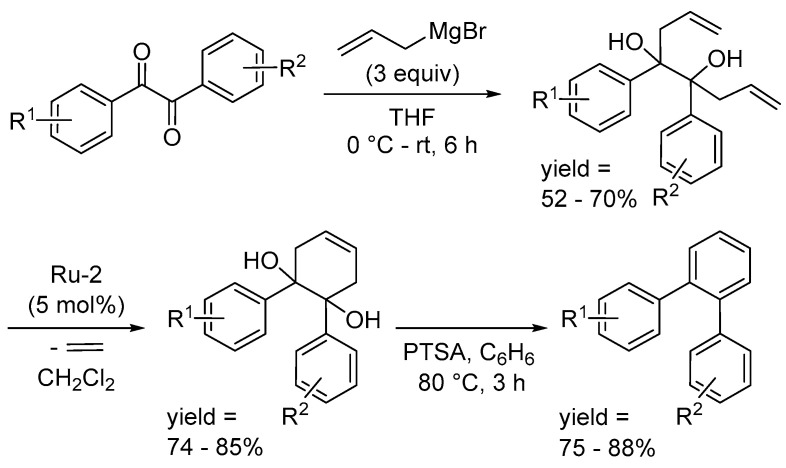
RCM approach for the synthesis of *o*-terphenyls.

Dash and co-workers demonstrated that *o*-terphenyls can be synthesized from easily accessible substituted benzils. The proposed synthetic pathway involves RCM for the synthesis of a tricyclic skeleton and subsequent aromatization step. It enables the synthesis of symmetrical and unsymmetrical *o*-terphenyls containing both electron-rich and electron-deficient groups (Scheme 43) [57].

**Scheme 44:**
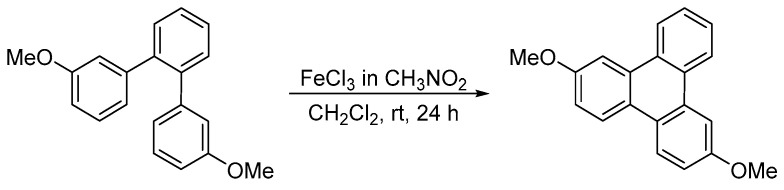
Synthesis of triphenylene derivatives from *o*-terphenyls.

Electron-rich *o*-terphenyls were successfully transformed to triphenylenes via the Sholl reaction (Scheme 44).

### 4.3. Synthesis of Aromatic Ring in Complex Molecules

**Scheme 45:**
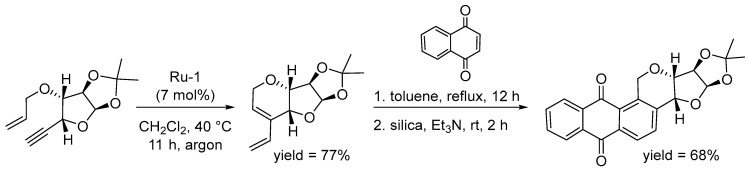
Sequence RCEM/cycloaddition/oxidation in the synthesis of sugar–oxasteroid–quinone hybrids.

Kaliappan reported the synthesis of a new class of sugar–oxasteroid–quinone hybrid molecules via a sequential enyne-metathesis/Diels–Alder cycloaddition/aromatization (Scheme 45) [58].

The obtained Diels–Alder cycloadducts were unstable, and their tendency to undergo aromatization (aerobic oxidation on silica gel) was observed. For maximum aromatization efficiency, the crude cycloaddition product was immediately treated with trimethylamine and silica gel. The method yielded a library of sugar–oxasteroid–quinone hybrids.

**Scheme 46:**
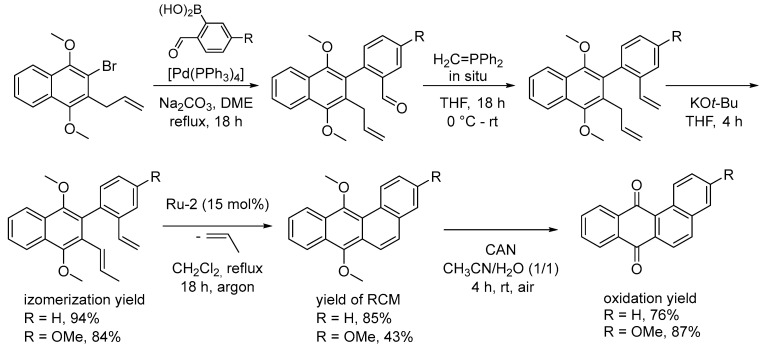
Key steps in the synthesis of the angucycline core.

De Koning and co-workers proposed novel methods for the assembly of benz[*a*]anthracenes and related compounds [59]. Their strategy involves the Suzuki–Miyaura cross-coupling, isomerization, and ring-closing metathesis. The RCM step in this case results in the formation of an aromatic ring, which can be easily oxidized to the corresponding quinone (Scheme 46).

**Figure 5:**
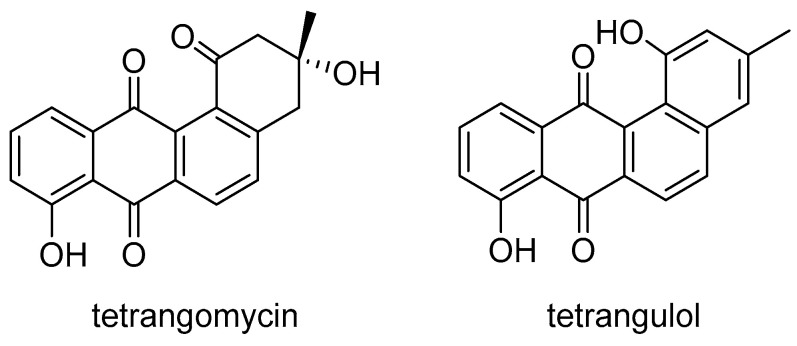
Structures of exemplary angucyclines.

Examples of structures of antibiotics from the angucycline group are shown in Figure 5.

**Scheme 47:**
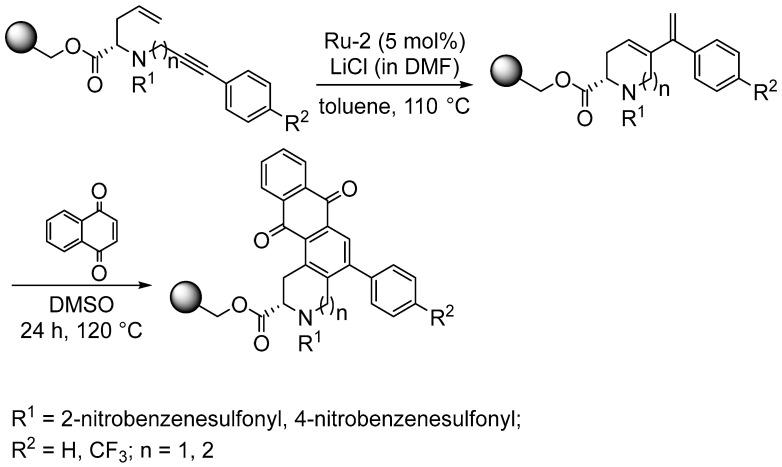
Key steps in solid-phase synthesis of tetrahydroisoquinolines.

The solid-phase synthesis of numerous polycyclic tetrahydroisoquinolines and tetrahydro-benzo[*d*]azepines starting from Wang resin-immobilized allylglycine was reported by Soural [60]. Subsequent Fmoc cleavage, sulfonylation with nitrobenzenesulfonyl chlorides, and Mitsunobu alkylation with phenylalkynols afforded corresponding enynes. Further ring-closing metathesis, cycloaddition with functionalized 1,4-naphthoquinones, and finally spontaneous aromatization enables the formation of a variety of tetrahydroisoquinolines and tetrahydrobenzo[d]azepines (Scheme 47). The proposed further functionalization of the obtained derivatives by heterocyclization enables the rapid synthesis of relevant bioactive scaffolds.

**Scheme 48:**
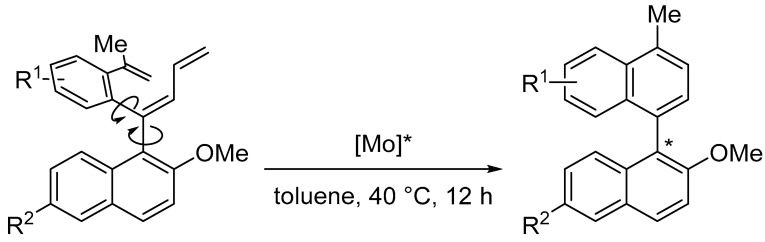
Asymmetric synthesis of binaphthalene atropisomers from stereodynamic trienes.

Recently, Sparr and co-workers reported on the great potential of metathesis in the atroposelective formation of arenes [61]. The authors demonstrated that, in the presence of enantiopure molybdenum metathesis catalysts, stereodynamic trienes were enantioselectively converted into the corresponding binaphthalene atropisomers (Scheme 48).

**Figure 6:**
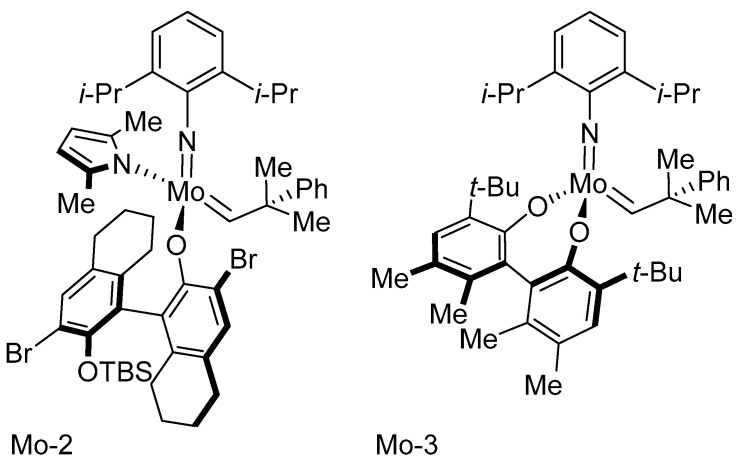
Commercially available chiral molybdenum alkylidene complexes tested in atroposelective synthesis of binaphthalenes.

In the presence of commercially available molybdenum catalysts (Figure 6), enantioselectivity, e.r. = 83:17, was observed for the model triene.

**Scheme 49:**
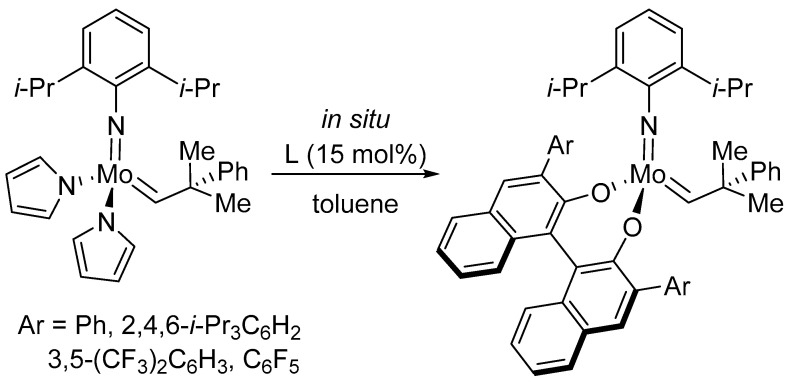
In situ generation of chiral, non-racemic molybdenum binaphthol complexes.

In order to optimize the yield and enantioselectivity, tests were carried out in the presence of a series of catalysts obtained in situ by reacting a dipyrrolyl molybdenum precursor with binaphthol ligands differing in stereoelectronic properties (Scheme 49).

**Figure 7:**
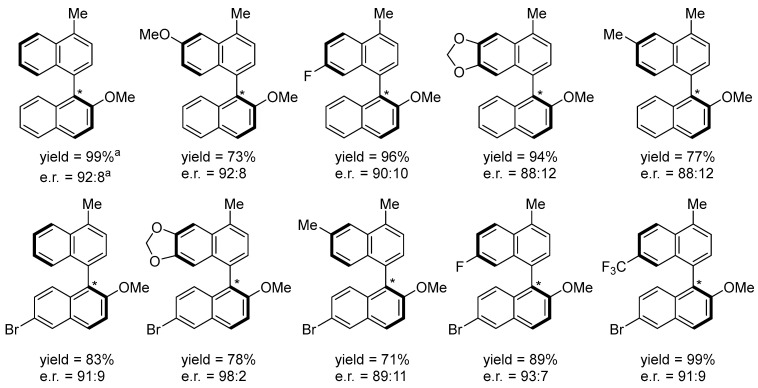
The scope of synthesized binaphthyl atropisomers.

It was shown that the most enantioselective catalyst was the binaphthyl complex containing pentafluorophenyl substituents. Under optimized conditions, a variety of conformationally dynamic triene substrates were efficiently and enantioselectively converted into binaphthyl atropisomers (Figure 7).

**Scheme 50:**
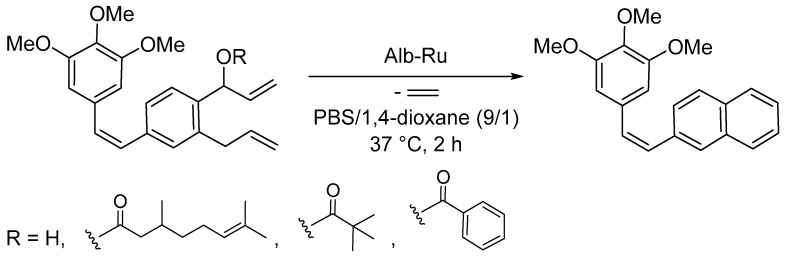
Naphthyl analogues of combretastatin synthesized by sequential ring-closing metathesis and aromatization.

**Figure 8:**
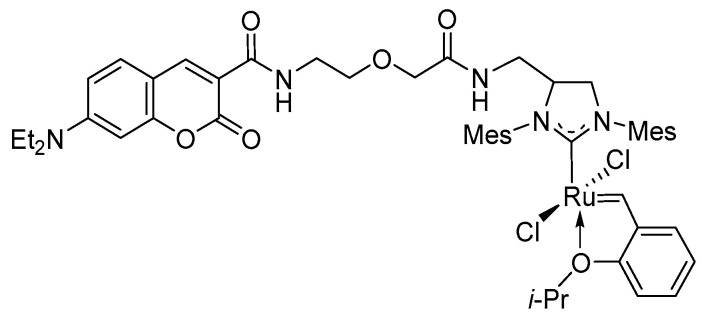
Glycosylated Hoveyda–Grubbs-type artificial metalloenzyme (Alb-Ru).

Finally, Tanaka and co-workers discussed the possibility of using RCM for the in vivo synthesis of cytostatic agents [62]. Thus, cytostatic agents, naphthyl analogues of combretastatin, can be synthesized by the sequential ring-closing metathesis and aromatization of relevant prodrugs (Scheme 50). It was shown that, in the presence of glycosylated Hoveyda–Grubbs-type artificial metalloenzyme Alb-Ru (Figure 8) and in biologically relevant concentrations of prodrugs (dienes), the proposed reaction sequence proceeds readily for all ester-based prodrugs, with pivalate being the most active. Therapy against cancer-cell growth was studied in cellulo and in vivo in mice.

The promising results show new possibilities for the use of artificial metalloenzymes and metathesis cyclization in pro-drug therapy.

### 4.4. Synthesis of Polyarenes

**Scheme 51:**
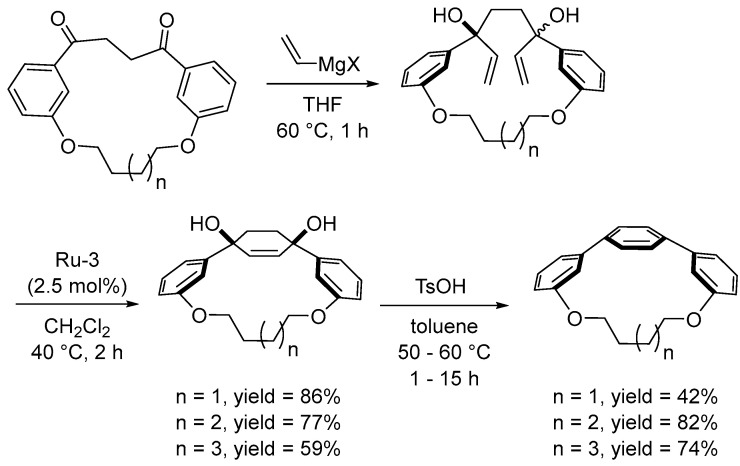
RCM and aromatization step in the synthesis of *m*-terphenylophanes.

Cycloparaphenylenes have gained the interest of many research groups in the hope that these systems can be used for the bottom-up chemical synthesis of carbon nanotubes [63,64]. A series of papers on the synthetic strategy that employs 1,4-diketo-bridged macrocycle as a precursor to a strained 1,4-arene-bridged (bent *para*-phenylene) macrocycle were reported by Merner and co-workers [65,66,67,68]. The described strategy involves a metathesis cyclization step, followed by aromatization (Scheme 51). Efficient metathesis cyclization was observed only for the *syn* diastereomer.

**Scheme 52:**
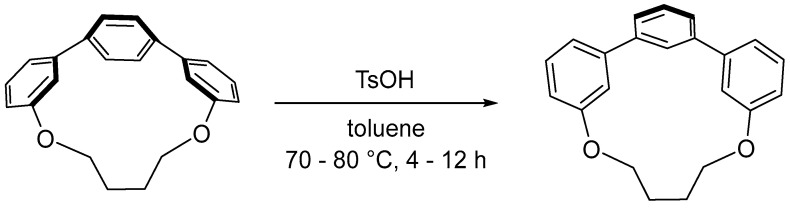
Strain-relief-driven rearrangement of *p*-terphenylophane to *m*-terphenylophane.

The aromatization reaction may be accompanied by a rearrangement of the desired *p*-terphenylophane to *m*-terphenylophane, which was observed for the arene-bridged macrocycle (Scheme 52) [66,67]. Such a strain-relief-driven rearrangement is not observed for n = 2 (Scheme 51). The selectivity of the aromatization process strongly depends on the reaction conditions and the reagents used. It was found that, in the presence of the Burgess reagent, the reaction selectively leads to the expected product.

**Scheme 53:**
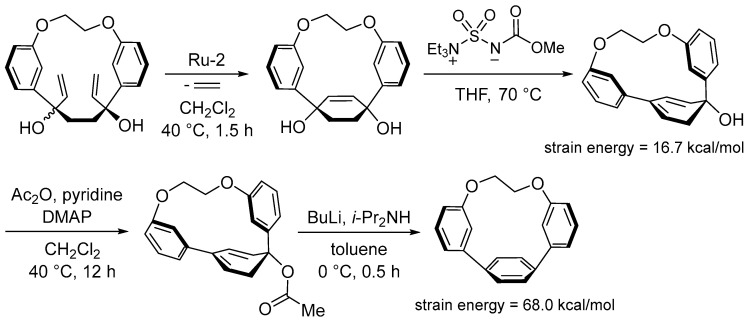
Merner’s strategy to obtain a strained aromatic ring.

Merner’s strategy enabled the synthesis of a *p*-terphenyl-based macrocycle containing a *p*-phenylene unit with 42.6 kcal/mol of strain energy [64]. The sequence uses metathesis cyclization (Scheme 53), but aromatization is crucial to obtain a stretched ring. It involves the use of iterative elimination reactions of the two hydroxyl groups present in the macrocyclic precursor [66,67].

**Scheme 54:**
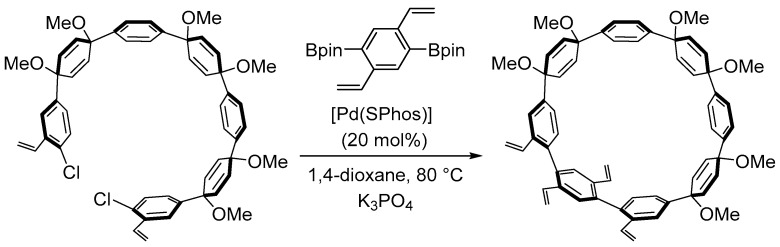
Synthesis of macrocycles via Suzuki cross-coupling.

The synthesis of double-stranded aromatic macrocycles remains a challenge. Jasti and co-workers developed a reductive aromatization/ring-closing metathesis sequence for the synthesis of aromatic belt fragments (Scheme 54) [69].

**Scheme 55:**
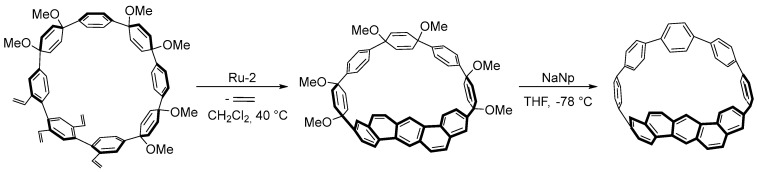
RCM/reductive aromatization sequence.

For the macrocycle presented in Scheme 55, the sequence starting from RCM was used in order to avoid potential problems of interaction of strong reductant (sodium naphthalenide) with styrene functionality. RCM proceeded readily in the presence of Ru-2 in dichloromethane at 40 °C. No side-products were observed. As shown in Scheme 55, the RCM strategy used leads to the formation of aromatic rings. A final aromatization step was made by subjecting macrocycle to sodium naphthalenide at −78 °C.

**Scheme 56:**
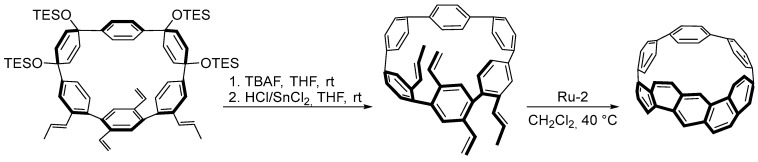
Reductive aromatization/RCM sequence in the synthesis of highly strained aromatic belt fragment.

The proposed strategy, which is a combination of reductive aromatization and ring-closing metathesis, was tested for the synthesis of smaller, more strained belts. In this case, the order of steps was changed, and the reductive aromatization was carried out prior to the RCM (Scheme 56). Deprotection of hydroxy groups, followed by reductive aromatization under mild conditions, yields strained ring. In the last step, the RCM enabled the formation of a strongly strained fragment of aromatic belt. Jasti demonstrated that the RCM reaction leading to the formation of aromatic rings can be carried out in highly strained systems, without undesirable side-processes [69].

**Scheme 57:**
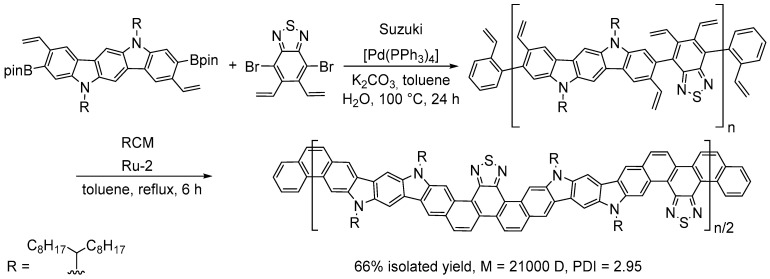
Synthesis of donor–acceptor ladder polymer by the sequence of Suzuki polycondensation and ring-closing olefin metathesis.

Fang and co-workers proposed efficient synthesis of donor–acceptor ladder polymer by the sequence of Suzuki polycondensation and ring-closing olefin metathesis (Scheme 57) [70]. The authors assumed that the use of RCM as a ladderization step, which, in this case, is also an aromatization step, will afford the desired ladder polymer in good yield and without structural defects.

Preliminary tests of polymer properties were performed. As expected, the rigid coplanar nature of the ladder polymer was found to exhibit improved physicochemical properties that were not observed with non-ladder type analogs.

## 5. Summary and Outlook

The synthesis of aromatic compounds using olefins and enyne metathesis employs several previously described strategies. The synthesis of fused aromatic compounds can be accomplished directly through diene or enyne RCM. Other strategies include a cyclization step via RCM, followed by aromatization, which can be implemented by oxidation or elimination of suitable leaving groups. Finally, enyne RCM or ene-yne CM allows the formation of conjugated dienes that can undergo successive cyclization, mainly through Diels–Alder cycloaddition, and aromatization. Based on the results described and previous reports, it seems that further progress is possible, particularly in the aromatization step. The use of (spontaneous) aromatization through aerobic oxidation eliminates the need for strong oxidants, which can expand the reaction scope and/or improve the yields achieved. A fascinating idea is to use RCM for the synthesis of atropisomers, using metathetic cyclization of conformationally dynamic trienes. The development of the method may open up a new area of application of metathetic cyclization in the asymmetric synthesis of aromatic compounds. Exciting reports have been made on the syntheses of macrocyclic aromatic strips with high ring strain. The efficient synthesis of linear ladder polymers with a stiff coplanar structure has been achieved using the strong thermodynamic driving force of aromatization. Due to the stiffened structure, such polymers exhibit improved physicochemical properties compared to non-ladder analogues. Finally, the possibility of using metathesis/aromatization sequences in the synthesis of prodrugs is described. This opens up a new and poorly explored area of research.

The survey clearly shows that the field of research will continue to develop in the coming years. In particular, material and medicinal chemistry, in the broadest sense, remain an important and underexplored area of research.

## Data Availability

Not applicable.
